# Vulnerability of Evacuees Having No One to Consult after the Fukushima Nuclear Disaster: The Fukushima Health Management Survey

**DOI:** 10.3390/ijerph181910075

**Published:** 2021-09-25

**Authors:** Naoko Horikoshi, Masaharu Maeda, Mayumi Harigane, Hajime Iwasa, Michio Murakami, Maho Momoi, Saori Goto, Seiji Yasumura

**Affiliations:** 1 Radiation Medical Science Center for the Fukushima Health Management Survey, Fukushima Medical University, Fukushima 960-1295, Japan; masagen@fmu.ac.jp (M.M.); harigane@fmu.ac.jp (M.H.); hajimei@fmu.ac.jp (H.I.); michio0923@gmail.com (M.M.); maho-m@fmu.ac.jp (M.M.); saori-g@fmu.ac.jp (S.G.); yasumura@fmu.ac.jp (S.Y.); 2 Department of Public Health, Fukushima Medical University School of Medicine, Fukushima 960-1295, Japan; 3 Department of Disaster Psychiatry, Fukushima Medical University School of Medicine, Fukushima 960-1295, Japan; 4 Department of Health Risk Communication, Fukushima Medical University School of Medicine, Fukushima 960-1295, Japan

**Keywords:** Great East Japan Earthquake, help-seeking, high-risk approach, nuclear disaster, social networks

## Abstract

After the accident at the Fukushima nuclear power plant in 2011, caused by the Great East Japan Earthquake, some evacuees had no one to consult despite many local care providers offering assistance. This study identified the characteristics of individuals who did not receive consultations and the relevant determinants, and proposed the available measures to address this issue. Altogether, 32,699 participants aged 16 years or older and residing in the disaster area at Fukushima were surveyed. Those with no one to consult showed a significantly higher prevalence of psychological distress (16.2%, *p* < 0.001) and drinking problems (21.5%, *p* < 0.001). Multivariate analysis revealed that these behaviors were associated with the middle age group (i.e., 40–64 years old) (odds ratio [OR]: 1.30; 95% confidence interval [CI]: 1.16–1.46), men (OR = 2.46; 95% CI, 2.27–2.66), bad financial circumstances (OR = 2.11; 95% CI, 1.96–2.27), and living alone (OR = 1.53; 95% CI, 1.39–1.68). This research verifies that people with such characteristics were more likely to be isolated and vulnerable to psychiatric problems, such as depression. We suggest that it is integral for local care providers to recognize those who have no one to consult and provide targeted support.

## 1. Introduction

After the nuclear accident following the Great East Japan Earthquake in March 2011, evacuees in the Fukushima Prefecture were forced into long-term and widespread evacuation. Many evacuees were separated from their communities and families, which affected them both physically and psychologically. After the Chernobyl disaster, evacuees experienced mental distress, which developed into serious health problems [1]. Since FY2011 (fiscal year; from 1 April to 30 March) [2,3], Fukushima Prefecture has commissioned the Fukushima Medical University to annually conduct a questionnaire survey titled “Mental Health and Lifestyle Survey.” The target population is approximately 210,000 people residing in the municipalities, including areas designated as evacuation order areas after the incident at the Fukushima Daiichi Nuclear Power Station. We evaluated the mental and physical health of the affected people and their lifestyles based on their responses and provided telephonic support to those at high risk [4,5]. We worked with different stakeholders and local health care workers, including public health nurses in the affected municipalities, and other health care resources, such as the Fukushima Center for Disaster Mental Health, to build a support system. Although many support organizations implemented various measures, evacuees who were alone generally had no one to consult, even long after the disaster. Social ties are integral for mental health [6], and their influence is increasingly highlighted by local care providers. Furthermore, limited social relationships can be linked to isolation and more serious outcomes, such as suicidal behavior [7,8], and are essential for post-disaster mental health support. 

There are two major conceptual frameworks for examining “having no one to consult” in such a scenario. The first framework is the lack of social networks. Numerous studies were conducted on social networks and related terms. Wang [9] proposed five conceptual domains for social networks and other related terms regularly used in the research literature on mental health: “social network quantity,” “social network structure,” “social network quality,” “appraisal of relationships (emotional),” and “appraisal of relationships (resources).” Accordingly, we define “having no one to consult” as a lack of “social network quantity” and “social network quality.” Moreover, “having no one to consult” is considered to be a result of a lack of intention or inability to seek help from others. Help-seeking behavior among patients with depression has been studied in clinical settings [10], and the stigma and discrimination often associated with psychiatric illness and its treatment could be a significant psychological barrier [11]. Poor help-seeking skills can result in detrimental health outcomes even though there is no firm definition of help-seeking. Considering different mental health difficulties as a by-product of major disasters, whether affected people can significantly seek help from others to resolve their difficulties should be a public health priority. 

We considered that the people impacted by the accident at the nuclear power plant who had no one to consult were more likely to be a high-risk population. The purpose of this study was to elucidate the characteristics of “people who have no one to consult” and the related risk factors leading to various health problems among them. Efficient measures to inculcate help-seeking skills during major disasters were discussed as well.

## 2. Materials and Methods

### 2.1. Participants

**Figure 1:**
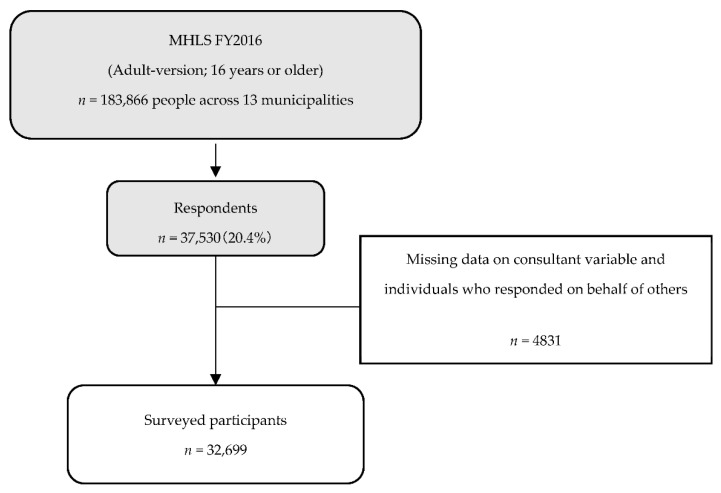
Selection of participants. MHLS: Mental Health Lifestyle Survey; FY: Fiscal Year.

The data used in this study were obtained from the “Mental Health and Lifestyle Survey” administered by Fukushima Medical University and commissioned by Fukushima Prefecture in FY2016. This study included approximately 180,000 people aged 16 years and above living in municipalities encompassing evacuation order areas. The details of these are provided in previous papers [2,3]. The number of respondents was 37,530 (20.4%, response rate), and the analysis included 32,699 people, since those reporting incomplete data and those who responded on behalf of other individuals were excluded (Figure 1).

### 2.2. Study Variables

The outcome variable was examined via the following question: “Do you have someone, or go to a specific institution, for consultation when experiencing a mental or physical problem following The Great East Japan Earthquake?” We defined those who answered “No” as people having no one to consult (HNC) and those who answered “Yes” as people having someone to consult (HSC). The demographic characteristics analyzed were age groups at the time of survey (i.e., 16–39 years, 40–64 years, and ≥65 years), sex, employment status, financial circumstances, residence (i.e., inside/outside Fukushima Prefecture), living alone, medical histories (i.e., presence/absence of a history of mental illness and lifestyle-related diseases), and disaster-related variables (i.e., presence/absence of separation from family). Psychological distress was inspected using the K6, a 6-item, self-administered, standardized screening instrument for non-specific psychological distress during the past 30 days [12,13]. A cutoff point of 13 was employed in this study, which was also frequently used in previous studies [14,15]. Drinking problems were assessed, among those candidates who were more than 20 years of age and drank alcohol at least once a month, using the CAGE, which was a valid and effective screening tool for alcoholism [16]. This tool comprised four questions, and based on previous research [17], participants with two or more affirmative responses were classified as alcoholics. 

### 2.3. Statistical Analysis

We excluded missing HNC-related data and a respondent who did not fulfill the inclusion criteria. First, descriptive statistics for socio-demographic characteristics, medical histories, disaster-related variables, psychological distress, and drinking problems in the study population were calculated to explore determinants associated with the HNC group. Subsequently, these variables were compared in the presence and absence of the HNC group using chi-square tests or t-tests.

We categorized the age of the respondents into three groups: 16–39 years, 40–64 years, and ≥65 years. The scores of the measures are expressed as mean (M) and standard deviation (SD). Thereafter, we studied factors associated with the HNC group using multivariate logistic regression analysis by age group, excluding variables concerned with the history of mental illness/lifestyle-related diseases, psychological distress, and drinking problems. A probability value of *p* < 0.05 was set to indicate statistical significance, and Statistical Package for the Social Sciences (SPSS) for Windows (version 27; Armonk, NY, USA) was used for all analyses.

### 2.4. Ethical Considerations 

This study was approved by the Ethics Review Committee of Fukushima Medical University (No. 2020-239). We explained, in writing, to the participants that the responses would not be published in any form that discloses their identity. Participants’ responses to the self-administered questionnaires were considered as their consent to participate.

## 3. Results

Table 1 presents the associations between participant demographics and the HNC group. Altogether, 3650 respondents (11.2%) were found in the HNC group, and the proportion of HNC was higher among males than females and, in the 40–64 age group, higher among the three age groups. Moreover, HNC was associated with employment, a bad financial situation, residing outside Fukushima Prefecture, living alone, separation from family, and a history of mental health/lifestyle-related diseases. Particularly, we found significant differences between psychological distress (K6 ≥ 13) (16.2%, *p* < 0.001) and drinking problems (CAGE ≥ 2) (21.5%, *p* < 0.001).

Table 2 presents the association of the HNC group with the characteristic variables among the three age groups. For all age groups, the HNC group was significantly associated with males, employment, bad financial situation, living alone, having psychological distress, and having drinking problems. Conversely, living outside the Fukushima Prefecture was correlated with the HNC groups aged 40–64 years (19.1%, *p* = 0.002) and ≥65 years (14.6%, *p* < 0.001). However, the history of mental illness was only associated with the ≥65 years age group (11.2%, *p* < 0.001), and the history of lifestyle-related diseases was linked to 16–39 years and 40–64 years age groups (19.1%, *p* < 0.001, 63.5%, *p* < 0.001, respectively). In addition, separation from family was significantly associated with the 40–64 and ≥65 years age groups (37.8%, *p* < 0.008, 32.5%, *p* = 0.001, respectively). Furthermore, for psychological distress, the younger the age group, the higher the proportion of members in the HNC group (i.e., 25.9%, 17.3%, and 12.4% for 16–39, 40–64, and ≥65 years age groups, respectively).

Table 3 shows the results of the logistic regression analyses with the members of the HNC group as the dependent variable by the total and age groups. As for age, the 40–64 years age group had significantly higher odds ratios (ORs) than those aged 16–39 years (OR = 1.30; 95% confidence interval [CI], 1.16–1.46). Notably, men (OR = 2.46; 95% CI, 2.27–2.66 for total), bad financial circumstances (OR = 2.11; 95% CI, 1.96–2.27), and living alone (OR = 1.53; 95% CI, 1.39–1.68) were significantly associated with HNC across all participant and age groups. Employment was only linked to the ≥65 years age group (OR =1.26; 95% CI, 1.09–1.45), and living outside of the Fukushima Prefecture was associated with the 40–64 and ≥65 years age groups (OR = 1.24; 95% CI, 1.06–1.44; and OR = 1.59; 95%CI, 1.36–1.87, respectively). No significant associations were found between the separation from family and the HNC group, irrespective of the age group.

## 4. Discussion

This study examined the characteristics and factors related to HNC (defined as people having no one to consult) in the Fukushima Prefecture. The HNC constituted about 11.2% of the selected candidates but reported a significantly higher prevalence of psychological distress and drinking problems, consistent with the hypothesis given above. For psychological distress, the younger the age, the higher the proportion of members in the HNC group. Poor mental health, such as depression, abetted poor connectivity (i.e., social networks) [18,19]. Concerns about health conditions and confidentiality were considered to result from public stigma and self-stigma [11]. It might have been a common reaction for people to suffer from mental instability due to evacuation, and we considered measures that reduced stigma and enabled people not to hesitate in seeking help when problems emerged. For instance, establishing social relationships and functions was important so that people in the society as a whole could easily consult each other. Such endeavors included improving the consultation functions at schools and workplaces and building connections with people even before the disaster occurred. Measures to dispel social stigma, including anti-stigma campaigns, were vital in disaster settings, considering that the intense social stigma toward people with psychiatric problems in Japan could reduce an individual’s capacity to seek consultation [20]. Furthermore, the prevalence of drinking problems was higher in the 40–64 age group than in others, and middle-aged drinkers of the HNC group should be specifically targeted. These factors implied that the HNC group offered a noteworthy perspective for high-risk groups in the aftermath of a disaster. We also discovered that the distribution of members within the HNC group differed according to the age group, and middle-aged individuals (i.e., people in the 40–64 years age group) were higher in number compared to other age groups. Thus, we analyzed age groups both collectively and individually to determine factors related to the HNC group.

Interestingly, common factors among the three age groups were being male, bad financial circumstances, and living alone. Psychological distress and drinking problems were significantly associated with the HNC group as well as all other age groups. The outcomes of past studies were diverse; one showed that the higher the age, the more positive the help-seeking attitude [21]; while another signified that the middle age group was most likely to seek advice [22]. Currently, it is unclear whether there is an age difference associated with help-seeking attitudes and to what extent this is the case [23]; culture-sensitive issues might impact the results. Furthermore, there was no positive correlation of HNC with age in this study. Likewise, men aged 40–64 years with psychological distress were significantly associated with the HNC group in an epidemiological study focusing on risk factors for depression [18]. With regard to mental health prevention, further studies examining the impact of social networks with a focus on middle aged people should be conducted. 

Various ideologies, norms, and gender roles made men become part of the HNC group [24]. Men were less inclined to develop social affiliations and were less likely to seek help from social support groups than women were [25,26]. They also had less contact with their families and neighbors during their lifetime [27,28]. Notably, studies concentrating on depression revealed that men were more hesitant to seek professional mental health care [29,30]. Therefore, we believe that the HNC group may be affected by gender differences and that gender-sensitive support measures are needed. Consistent with previous studies [31], the HNC group was associated with bad financial circumstances among all age groups. As in the previous study, people with bad financial circumstances were not likely to ask for help [32]. Furthermore, for all age groups, living alone was associated with the HNC group. The findings differed from those of past studies [33], which claimed that people living alone were easier to isolate, and paradoxically, they would possibly seek help. Nevertheless, some findings suggested that people who lived alone had more negative attitudes toward seeking help [34]. As the number of people living alone is increasing globally and society is drastically changing [35], people living alone and belonging to the HNC group must be prioritized.

In the elderly age group (≥65 years), the HNC group was significantly associated with employment. The fact that elderly individuals continued to be employed may mean that they had a work-centered life [27] and were unable to rely on social resources, such as family, friends, and neighbors. Furthermore, we found that the association between those residing outside Fukushima Prefecture at the time of the survey and the HNC group was significant among middle-aged and older people aged ≥40 years. After the Fukushima nuclear disaster, many evacuees were forced to evacuate their hometowns and moved more than four times during the first year after the disaster [3,36]. Studies have shown that relocation after a disaster increases the risk of psychological problems among evacuees [37]; the higher the age, the stronger the feeling of separation from one’s hometown becomes. Consequently, evacuees’ links to old social networks and connections may have been disconnected, thus forcing them to become part of the HNC group.

In this study, we used large-scale data from the Mental Health and Lifestyle of the Fukushima Health Management Survey to perform an exploratory analysis of factors associated with the HNC group. However, this study has several limitations. First, it was not possible to establish a cause-and-effect relationship because this study was cross-sectional by design. We recommend that more longitudinal studies should be conducted to establish causal relationships. In addition, the response rate for this survey was approximately 20%, which might not be representative of the target population. Some reasons for such a low response rate include lack of time to complete the questionnaire for the participants and the burden on them due to the questionnaire’s volume [38]. On the other hand, a study examining the possible response bias in this survey showed that non-respondents had significantly higher rates of psychological distress than respondents, and that there was a correlation between poor social support and low response rates [38]. Therefore, in the present study, it is possible that the proportion of the HNC group among the non-respondent group was higher than that among the respondent group, and that such response bias might affect the results. In spite of these limitations, we believe that the findings of this survey suggest the importance of concentrating on the HNC group for local care providers who provide direct services to evacuees. It is easy for local care providers to ask evacuees, “Do you have someone to consult?” and we think it is integral to relay this message to the HNC group so that local care providers can gradually become their advisors.

## 5. Conclusions

In this study, we showed that the HNC group was associated with a significantly higher prevalence of psychological distress and drinking problems, as well as with the middle-aged group (i.e., 40–64 years old), men, bad financial circumstances, and living alone among the affected people in Fukushima Prefecture. As in previous studies, our study revealed that concentrating on the HNC group was crucial for maintaining and developing individuals’ health conditions, especially mental health. Therefore, local care providers need to understand the vulnerability of the evacuees in the HNC group and provide early support to them as they are high-risk individuals.

## Figures and Tables

**Table 1:** Characteristics of participants.

	Overall Participants	HNC	HSC	*p*
		(*n* = 3650)	(*n* = 29,049)	
Age				
Mean (SD)	61.2 (17.4)	60.2 (16.0)	61.4 (17.5)	*p* < 0.001
16–39 years	4740 (14.5)	473 (13.0)	4267 (14.7)	*p* < 0.001
40–64 years	10,893 (33.3)	1446 (39.6)	9447 (32.5)	
≥65 years	17,066 (52.2)	1731 (47.4)	15,335 (52.8)	
Sex				
Men	14,698 (44.9)	2350 (64.4)	12,348 (42.5)	*p* < 0.001
Women	18,001 (55.1)	1300 (35.6)	16,701 (57.5)	
Employment status				
Employed	11,458 (36.9)	1522 (43.9)	9936 (36.1)	*p* < 0.001
Unemployed	19,567 (63.1)	1945 (56.1)	17,622 (63.9)	
Financial circumstances				
Good	20,975 (66.2)	1761 (49.5)	19,214 (68.3)	*p* < 0.001
Bad	10,721 (33.8)	1796 (50.5)	8925 (31.7)	
Residence				
Inside Fukushima prefecture	27,920 (85.4)	3002 (82.2)	24,918 (85.8)	*p* < 0.001
Outside Fukushima prefecture	4779 (14.6)	648 (17.8)	4131 (14.2)	
Living alone				
Yes	4868 (15.4)	773 (22.2)	4095 (14.6)	*p* < 0.001
No	26,739 (84.6)	2713 (77.8)	24,026 (85.4)	
Separation with family				
Yes	9945 (31.3)	1228 (34.8)	8717 (30.8)	*p* < 0.001
No	21,838 (68.7)	2296 (65.2)	19,542 (69.2)	
History of mental illness				
Yes	2971 (9.4)	392 (11.1)	2579 (9.2)	*p* < 0.001
No	28,593 (90.6)	3127 (88.9)	25,466 (90.8)	
History of lifestyle-related diseases				
Yes	20,018 (61.9)	2342 (65.0)	17,676 (61.5)	*p* < 0.001
No	12,328 (38.1)	1262 (35.0)	11,066 (38.5)	
K6				
<13	29,011 (93.2)	2911 (83.8)	26,100 (94.4)	*p* < 0.001
≥13	2104 (6.8)	562 (16.2)	1542 (5.6)	
CAGE				
<2	10,947 (85.5)	1322 (78.5)	9625 (86.5)	*p* < 0.001
≥2	1861 (14.5)	363 (21.5)	1498 (13.5)	

*n* (%), mean (SD), Chi-square test and *t*-test were used. HNC: People having no one to consult. HSC: People who have someone to consult.

**Table 2:** The association between the HNC group among the three age groups.

	16–39 Years			40–64 Years			≥65 Years		
	HNC	HSC	*p*	HNC	HSC	*p*	HNC	HSC	*p*
	(*n* = 473)	(*n* = 4267)		(*n* = 1446)	(*n* = 9447)		(*n* = 1731)	(*n* = 15,335)	
Sex									
Men	262 (55.4)	1403 (32.9)	<0.001	946 (65.4)	3936 (41.7)	<0.001	1142 (66.0)	7009 (45.7)	<0.001
Women	211 (44.6)	2864 (67.1)		500 (34.6)	5511 (58.3)		589 (34.0)	8326 (54.3)	
Employment status									
Employed	293 (62.6)	2335 (55.0)	0.002	898 (63.6)	5376 (57.9)	<0.001	331 (20.9)	2225 (15.9)	<0.001
Unemployed	175 (37.4)	1908 (45.0)		514 (36.4)	3905 (42.1)		1256 (79.1)	11,809 (84.1)	
Financial circumstances									
Good	211 (44.9)	2733 (64.4)	<0.001	707 (49.4)	6233 (66.9)	<0.001	843 (50.9)	10,248 (70.3)	<0.001
Bad	259 (55.1)	1508 (35.6)		724 (50.6)	3078 (33.1)		813 (49.1)	4339 (29.7)	
Residence *									
Inside	353 (74.6)	3232 (75.7)	0.592	1170 (80.9)	7947 (84.1)	0.002	1479 (85.4)	13,739 (89.6)	<0.001
Outside	120 (25.4)	1035 (24.3)		276 (19.1)	1500 (15.9)		252 (14.6)	1596 (10.4)	
Living alone									
Yes	102 (22.0)	619 (14.8)	<0.001	337 (24.0)	1219 (13.1)	<0.001	334 (20.7)	2257 (15.4)	<0.001
No	361 (78.0)	3571 (85.2)		1069 (76.0)	8073 (86.9)		1283 (79.3)	12,382 (84.6)	
Separation with family									
Yes	160 (34.1)	1304 (30.8)	0.136	536 (37.8)	3198 (34.2)	0.008	532 (32.5)	4215 (28.7)	0.001
No	309 (65.9)	2936 (69.2)		881 (62.2)	6142 (65.8)		1106 (67.5)	10,464 (71.3)	
History of mental illness									
Yes	55 (11.7)	392 (9.2)	0.082	153 (10.8)	969 (10.4)	0.609	184 (11.2)	1218 (8.4)	<0.001
No	415 (88.3)	3858 (90.8)		1259 (89.2)	8358 (89.6)		1453 (88.8)	13,250 (91.6)	
History of lifestyle-related diseases									
Yes	90 (19.1)	528 (12.4)	<0.001	908 (63.5)	5272 (56.1)	<0.001	1344 (78.9)	11,876 (78.7)	0.854
No	381 (80.9)	3723 (87.6)		521 (36.5)	4125 (43.9)		360 (21.1)	3218 (21.3)	
K6									
<13	346 (74.1)	3964 (93.4)	<0.001	1167 (82.7)	8746 (94.3)	<0.001	1398 (87.6)	13,390 (94.8)	<0.001
≥13	121 (25.9)	280 (6.6)		244 (17.3)	529 (5.7)		197 (12.4)	733 (5.2)	
CAGE									
<2	141 (81.5)	1275 (89.4)	0.002	576 (76.7)	3811 (85.4)	<0.001	605 (79.5)	4539 (86.7)	<0.001
≥2	32 (18.5)	151 (10.6)		175 (23.3)	649 (14.6)		156 (20.5)	698 (13.3)	

*n* (%), mean (SD), Chi-square test and *t*-test were used. HNC: People having no one to consult. HSC: People who have someone to consult. Residence*: Inside (Inside Fukushima prefecture). Outside (Outside Fukushima prefecture).

**Table 3:** The results of the multivariate logistic regression analysis regarding factors associated with having no one to consult according to age groups.

Variable	Total			16–39 years			40–64 years			≥65 years		
	(*n* = 32,699)			(*n* = 4740)			(*n* = 10,893)			(*n* = 17,066)		
	OR	95% CI	*p*	OR	95% CI	*p*	OR	95% CI	*p*	OR	95% CI	*p*
Age												
16–39 years (Ref)	1.00	-		-	-	-	-	-	-	-	-	-
40–64 years	1.30	1.16–1.46	0.001	-	-	-	-	-	-	-	-	-
≥65 years	1.01	0.90–1.15	0.818	-	-	-	-	-	-	-	-	-
Sex												
Women (Ref)	1.00	-		1.00	-	-	1.00	-	-	1.00	-	-
Men	2.46	2.27–2.66	<0.001	2.49	2.04–3.06	<0.001	2.57	2.26–2.91	<0.001	2.36	2.10–2.66	<0.001
Employment status												
Unemployed (Ref)	1.00	-		1.00	-		1.00	-		1.00	-	
Employed	1.11	1.02–1.21	0.014	1.10	0.90–1.36	0.360	1.01	0.89–1.15	0.837	1.26	1.09–1.45	0.001
Financial circumstances												
Good (Ref)	1.00	-		1.00	-		1.00	-		1.00	-	
Bad	2.11	1.96–2.27	<0.001	2.31	1.89–2.82	<0.001	2.04	1.81–2.29	<0.001	2.12	1.90–2.37	<0.001
Residence												
Inside Fukushima prefecture (Ref)	1.00	-		1.00	-		1.00	-		1.00	-	
Outside Fukushima prefecture	1.30	1.18–1.44	<0.001	1.01	0.80–1.28	0.921	1.24	1.06–1.44	0.006	1.59	1.36–1.87	<0.001
Living alone												
No (Ref)	1.00	-		1.00	-		1.00	-		1.00	-	
Yes	1.53	1.39–1.68	<0.001	1.49	1.16–1.92	0.002	1.61	1.39–1.87	<0.001	1.48	1.28–1.72	<0.001
Separation from family												
No (Ref)	1.00	-		1.00	-		1.00	-		1.00		
Yes	1.07	0.98–1.16	0.117	1.01	0.81–1.25	0.955	1.06	0.94–1.21	0.327	1.09	0.97–1.23	0.155

Note. Multivariate logistic regression was used to calculate odds ratios (ORs) and 95% confidence intervals (95% CI) after simultaneously controlling for independent variables.

## References

[1] World Health Organization (2006). Health Effects of the Chernobyl accident and Special Health Care Programs.

[2] Yasumura S., Hosoya M., Yamashita S., Kamiya K., Abe M., Akashi M., Kodama K., Ozasa K. (2012). Study Protocol for the Fukushima Health Management Survey. J. Epidemiol..

[3] Yabe H., Suzuki Y., Mashiko H., Nakayama Y., Hisata M., Niwa S., Yasumura S., Yamashita S., Kamiya K., Abe M. (2014). Mental health group of the Fukushima Health Management Survey. Psychological distress after the Great East Japan Earthquake and Fukushima Daiichi Nuclear Power Plant Accident: Results of a Mental Health and Lifestyle Survey through the Fukushima Health Management Survey in FY2011 and FY2012. Fukushima J. Med. Sci..

[4] Horikoshi N., Maeda M., Iwasa H., Momoi M., Oikawa Y., Ueda Y., Kashiwazaki Y., Onji M., Harigane M., Yabe H. (2020). The Usefulness of Brief Telephonic Intervention After a Nuclear Crisis: Long-Term Community-Based Support for Fukushima Evacuees. Disaster Med. Public Health Prep..

[5] Kashiwazaki Y., Maeda M., Yagi A., Fujii S., Takahashi N., Yabe H., Yasumura S., Abe M. (2016). Effectiveness of telephone-based intervention for people living in Fukushima Disaster Area: Fukushima Health Management Survey. Clin. Psychiatry.

[6] Nicholson N.R. (2012). A Review of Social Isolation: An Important but Underassessed Condition in Older Adults. J. Prim. Prev..

[7] Awata S., Seki T., Koizumi Y., Sato S., Hozawa A., Omori K., Kuriyama S., Arai H., Nagatomi R., Matsuoka H. (2005). Factors associated with suicidal ideation in an elderly urban Japanese population: A community-based, cross-sectional study. Psychiatry Clin. Neurosci..

[8] You S., Van Orden K.A., Conner K.R. (2011). Social connections and suicidal thoughts and behavior. Psychol. Addict. Behav..

[9] Wang J., Lloyd-Evans B., Giacco D., Forsyth R., Nebo C., Mann F., Johnson S. (2017). Social isolation in mental health: A conceptual and methodological review. Soc. Psychiatry Psychiatr. Epidemiol..

[10] Schnyder N., Panczak R., Groth N.S., Schultze-Lutter F. (2017). Association between mental health-related stigma and active help-seeking: Systematic review and meta-analysis. Br. J. Psychiatry.

[11] Clement S., Schauman O., Graham T., Maggioni F., Evans-Lacko S., Bezborodovs N., Morgan C., Rüsch N., Brown J.S., Thornicroft G. (2015). What is the impact of mental health-related stigma on help-seeking? A systematic review of quantitative and qualitative studies. Psychol. Med..

[12] Kessler R.C., Andrews G., Colpe L.J., Hiripi E., Mroczek D.K., Normand S.-L., Walters E.E., Zaslavsky A.M. (2002). Short screening scales to monitor population prevalences and trends in non-specific psychological distress. Psychol. Med..

[13] Furukawa T.A., Kawakami N., Saitoh M., Ono Y., Nakane Y., Nakamura Y., Tachimori H., Iwata N., Uda H., Nakane H. (2008). The performance of the Japanese version of the K6 and K10 in the World Mental Health Survey Japan. Int. J. Methods Psychiatr. Res..

[14] Kessler R.C., Barker P.R., Colpe L.J., Epstein J.F., Gfroerer J.C., Hiripi E., Howes M.J., Normand S.-L., Manderscheid R.W., Walters E.E. (2003). Screening for Serious Mental Illness in the General Population. Arch. Gen. Psychiatry.

[15] Suzuki Y., Yabe H., Yasumura S., Ohira T., Niwa S.-I., Ohtsuru A., Mashiko H., Maeda M., Abe M., on behalf of the Mental Health Group of the Fukushima Health Management Survey (2015). Psychological distress and the perception of radiation risks: The Fukushima health management survey. Bull. World Health Organ..

[16] Mayfield D., McLeod G., Hall P. (1974). The CAGE questionnaire: Validation of a new alcoholism screening instrument. Am. J. Psychiatry.

[17] Castells M.A., Furlanetto L.M. (2005). Validity of the CAGE questionnaire for screening alcohol-dependent inpatients on hospital wards. Rev. Bras. Psiquiatr..

[18] Kuriyama S., Nakaya N., Ohmori-Matsuda K., Shimazu T., Kikuchi N., Kakizaki M., Sone T., Sato F., Nagai M., Sugawara Y. (2009). Factors associated with psy-chological distress in a community-dwelling Japanese population: The Ohsaki Cohort 2006 Study. J. Epidemiol..

[19] Sone T., Nakaya N., Sugawara Y., Tomata Y., Watanabe T., Tsuji I. (2016). Longitudinal association between time-varying social isolation and psychological distress after the Great East Japan Earthquake. Soc. Sci. Med..

[20] Ando S., Yamaguchi S., Aoki Y., Thornicroft G. (2013). Review of mental-health-related stigma in Japan. Psychiatry Clin. Neurosci..

[21] MacKenzie C.S., Gekoski W.L., Knox V.J. (2006). Age, gender, and the underutilization of mental health services: The influence of help-seeking attitudes. Aging Ment. Health.

[22] Crabb R., Hunsley J. (2006). Utilization of mental health care services among older adults with depression. J. Clin. Psychol..

[23] Mackenzie C.S., Scott T., Mather A., Sareen J. (2008). Older Adults’ Help-Seeking Attitudes and Treatment Beliefs Concerning Mental Health Problems. Am. J. Geriatr. Psychiatry.

[24] Addis M.E., Mahalik J.R. (2003). Men, masculinity, and the contexts of help seeking. Am. Psychol..

[25] Gagné S., Vasiliadis H.-M., Préville M. (2014). Gender differences in general and specialty outpatient mental health service use for depression. BMC Psychiatry.

[26] Galdas P.M., Cheater F., Marshall P. (2005). Men and health help-seeking behaviour: Literature review. J. Adv. Nurs..

[27] Inoue M., Matsumoto S., Yamaoka K., Muto S. (2014). Risk of Social Isolation Among Great East Japan Earthquake Survivors Living in Tsunami-Affected Ishinomaki, Japan. Disaster Med. Public Health Prep..

[28] Kobayashi K.M., Cloutier-Fisher D., Roth M. (2009). Making meaningful connections: A profile of social isolation and health among older adults in small town and small city, British Columbia. J. Aging Health.

[29] Johnson J.L., Oliffe J.L., Kelly M.T., Galdas P.M., Ogrodniczuk J.S. (2011). Men’s discourses of help-seeking in the context of depression. Sociol. Health Illn..

[30] Oliffe J.L., Phillips M.J. (2008). Men, depression and masculinities: A review and recommendations. J. Mens Health.

[31] Murakami M., Kobayashi T., Oikawa Y., Goto S., Momoi M., Takebayashi Y., Ohira T., Yasumura S., Maeda M. (2021). Associ-ations of the COVID-19 pandemic with the economic status and mental health of people affected by the Fukushima Disaster using the Difference-in-Differences Method: The Fukushima Health Management Survey. SSM-Popul. Health.

[32] González H.M., Vega W.A., Williams D.R., Tarraf W., West B.T., Neighbors H.W. (2010). Depression care in the United States: Too little for too few. Arch. Gen. Psychiatry.

[33] Brandstetter S., Dodoo-Schittko F., Speerforck S., Apfelbacher C., Grabe H.-J., Jacobi F., Hapke U., Schomerus G., Baumeister S.E. (2017). Trends in non-help-seeking for mental disorders in Germany between 1997–1999 and 2009–2012: A repeated cross-sectional study. Soc. Psychiatry Psychiatr. Epidemiol..

[34] Coppens E., Van Audenhove C., Scheerder G., Arensman E., Coffey C., Costa S., Koburger N., Gottlebe K., Gusmão R., O’Connor R. (2013). Public attitudes toward depression and help-seeking in four European countries baseline survey prior to the OSPI-Europe intervention. J. Affect. Disord..

[35] Raymo J.M. (2015). Living alone in Japan: Relationships with happiness and health. Demogr. Res..

[36] Horikoshi N., Iwasa H., Kawakami N., Suzuki Y., Yasumura S. (2016). Residence-related factors and psychological distress among evacuees after the Fukushima Daiichi nuclear power plant accident: A cross-sectional study. BMC Psychiatry.

[37] Chen Y.-L., Lai C.-S., Chen W.-T., Hsu W.-Y., Wu Y.-C., Wang P.-W., Chen C.-S. (2011). Risk factors for PTSD after Typhoon Morakot among elderly people in Taiwanese aboriginal communities. Int. Psychogeriatr..

[38] Horikoshi N., Iwasa H., Yasumura S., Maeda M. (2017). The characteristics of non-respondents and respondents of a mental health survey among evacuees in a disaster: The Fukushima Health Management Survey. Fukushima J. Med Sci..

